# Biorefining of wheat straw: accounting for the distribution of mineral elements in pretreated biomass by an extended pretreatment–severity equation

**DOI:** 10.1186/s13068-014-0141-7

**Published:** 2014-10-14

**Authors:** Duy Michael Le, Hanne R Sørensen, Niels Ole Knudsen, Jan K Schjoerring, Anne S Meyer

**Affiliations:** DONG Energy, Kraftværksvej 53, DK-7000 Fredericia, Denmark; Center for BioProcess Engineering, Department of Chemical and Biochemical Engineering, Technical University of Denmark, DK-2800 Lyngby, Denmark; Plant and Soil Science Section, Department of Plant and Environmental Sciences, Faculty of Science, University of Copenhagen, DK-1871 Frederiksberg C, Copenhagen Denmark

**Keywords:** Hydrothermal pretreatment, Lignocellulose, pH, Minerals, Severity modeling

## Abstract

**Background:**

Mineral elements present in lignocellulosic biomass feedstocks may accumulate in biorefinery process streams and cause technological problems, or alternatively can be reaped for value addition. A better understanding of the distribution of minerals in biomass in response to pretreatment factors is therefore important in relation to development of new biorefinery processes. The objective of the present study was to examine the levels of mineral elements in pretreated wheat straw in response to systematic variations in the hydrothermal pretreatment parameters (pH, temperature, and treatment time), and to assess whether it is possible to model mineral levels in the pretreated fiber fraction.

**Results:**

Principal component analysis of the wheat straw biomass constituents, including mineral elements, showed that the recovered levels of wheat straw constituents after different hydrothermal pretreatments could be divided into two groups: 1) Phosphorus, magnesium, potassium, manganese, zinc, and calcium correlated with xylose and arabinose (that is, hemicellulose), and levels of these constituents present in the fiber fraction after pretreatment varied depending on the pretreatment-severity; and 2) Silicon, iron, copper, aluminum correlated with lignin and cellulose levels, but the levels of these constituents showed no severity-dependent trends. For the first group, an expanded pretreatment-severity equation, containing a specific factor for each constituent, accounting for variability due to pretreatment pH, was developed. Using this equation, the mineral levels could be predicted with R^2^ > 0.75; for some with R^2^ up to 0.96.

**Conclusion:**

Pretreatment conditions, especially pH, significantly influenced the levels of phosphorus, magnesium, potassium, manganese, zinc, and calcium in the resulting fiber fractions. A new expanded pretreatment-severity equation is proposed to model and predict mineral composition in pretreated wheat straw biomass.

## Introduction

In second-generation bioethanol production, instead of sucrose and starch, lignocellulosic agricultural waste streams are utilized as carbohydrate feed stocks, hence avoiding the ethical issues associated with turning food into fuel [1]. The regionally preferred type of lignocellulosic biomass depends on its local availability. In Europe, where wheat dominates (47% of the cereal crop production in Europe in 2012 was wheat [2]), wheat straw is therefore the most important biomass for second-generation bioethanol production.

A generalized linear process for producing second-generation bioethanol involves pretreatment to open the biomass structure, enzymatic hydrolysis of the cellulose and hemicellulose to form glucose and xylose, and fermentation of glucose, or of both glucose and xylose, into ethanol (most recently based on evolution of the xylose utilization rate in *Saccharomyces cerevisiae* by various techniques [3,4]). Recently, there has been increasing interest in expanding the concept from production of bioethanol to biorefineries, where the co-processing streams are used for production of various chemicals, building blocks or functional products, and/or other energy carriers [5-7].

Agglomeration, formation of deposits, slagging, fouling, and corrosion problems are well-described technological problems caused by mineral elements during thermochemical conversion of lignocellulosic biomass (other than wood) [8,9]. In the context of biorefining of lignocellulosic biomass, mineral elements may accumulate in certain streams, which may challenge the processing, and cause wear and tear of equipment. Alternatively, these may provide opportunities for recovery and recycling of scarce metals, and/or for creating novel high-value applications [10-12]. However, detailed information about the mineral content of the product streams is an overlooked subject in plant biomass biorefining, and information about the distribution of mineral elements in lignocellulosic biomass streams is sparse in the literature, despite such information being an important prerequisite for designing optimal biorefinery processes.

The current study was based on the hypothesis that the distribution of various minerals in wheat straw during hydrothermal pretreatment can be predicted and consequently controlled by the pretreatment conditions, such as temperature, treatment time, and pH during pre-soaking of the biomass. The objective of this study was to examine the levels of certain mineral elements in pretreated wheat straw in response to a systematic pretreatment campaign, and to evaluate how the behavior of these elements can be modeled.

## Results

### Composition and pretreatment factor analysis

**Figure 1:**
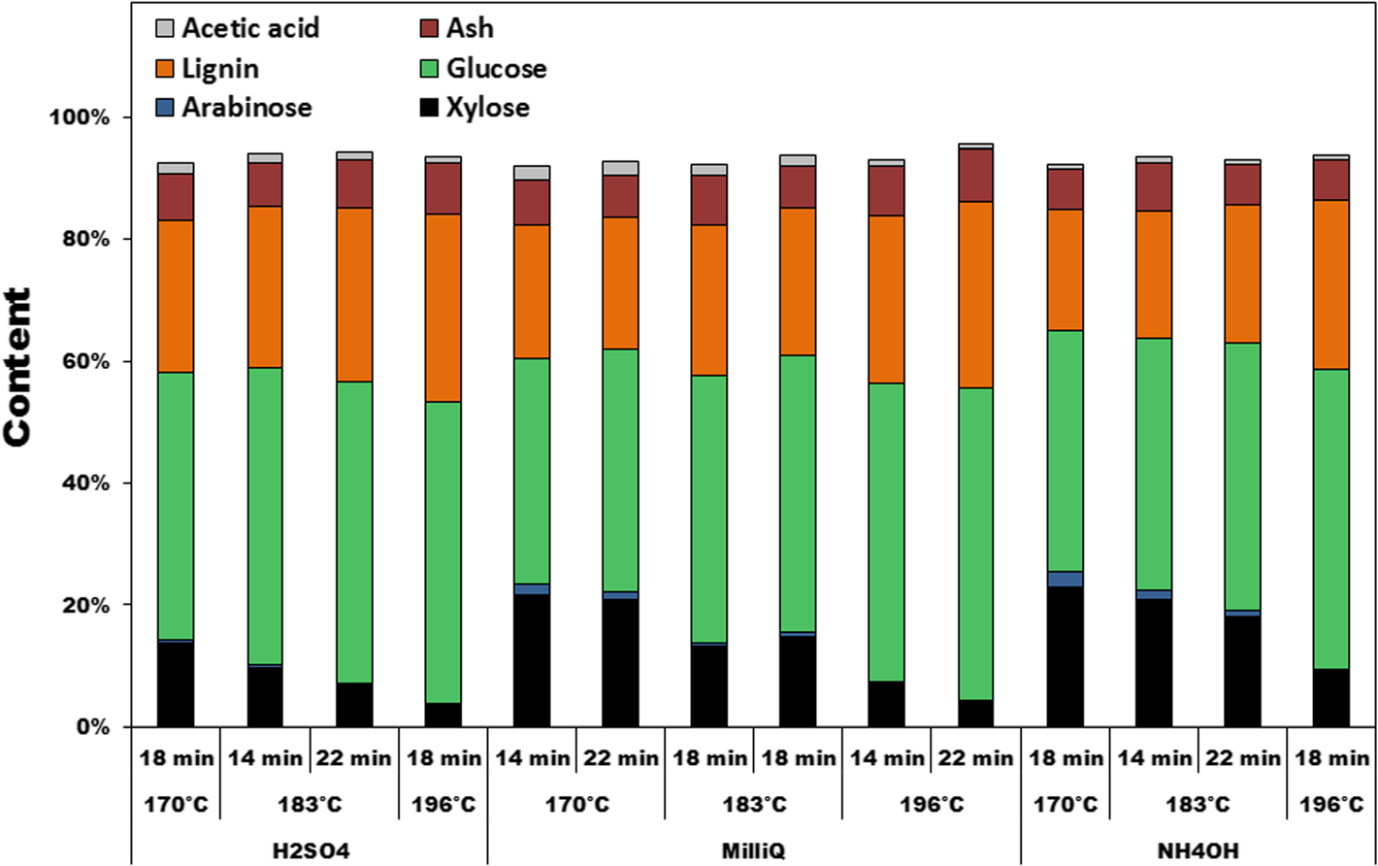
**Composition of the washed fiber fractions after hydrothermal pretreatment.**

Composition of the fiber fraction after hydrothermal pretreatment of wheat straw is shown in Figure 1, and summarized as content and recovery ranges for all biomass constituents measured in Table 1. Between 92% and 94% (by weight) of the dry matter of the biomass could be accounted for in the fiber fractions resulting from different pretreatment factor combinations (Figure 1). The three main components, xylose, glucose, and lignin, varied in relative concentration, but constituted between 80% and 86% of the dry matter of all the fiber fractions (calculated from data shown in Figure 1).

**Table 1:** **Content and recovery range of the components of the fiber fraction of pretreated wheat straw**

	**Content range (weight base)**	**Recovery range (weight base)**
Glucose	37 to 51%	74 to 98%
Lignin	20 to 31%	72 to 102%
Xylose	4 to 23%	10 to 74%
Arabinose	0 to 3%	1 to 61%
Silicon	11950 to 16554 ppm	56 to 89%
Potassium	864 to 2111 ppm	4 to 11%
Iron	774 to 1515 ppm	51 to 87%
Aluminum	649 to 1429 ppm	39 to 78%
Calcium	194 to 4143 ppm	3 to 73%
Magnesium	176 to 891 ppm	11 to 70%
Phosphorus	82 to 1769 ppm	3 to 72%
Zinc	31 to 102 ppm	22 to 88%
Manganese	10 to 101 ppm	7 to 93%
Copper	3 to 6 ppm	23 to 53%

Contents are given as percentages or ppm by weight of dry matter and recoveries are given as amount in fiber fraction in relation to amount in raw material (given in percent).

Although potassium was the most abundant mineral element in wheat straw before pretreatment, silicon was present in around 8 to 12-fold higher concentration than potasium after pretreatment (Table 1). Potassium was solubilized from the fiber fraction, and silicon hence became the most abundant mineral element in each fiber fraction. Silicon was particularly dominant following pretreatment with high temperatures and low pH, constituting up to 74% by mass of all mineral elements (data not shown).

**Figure 2:**
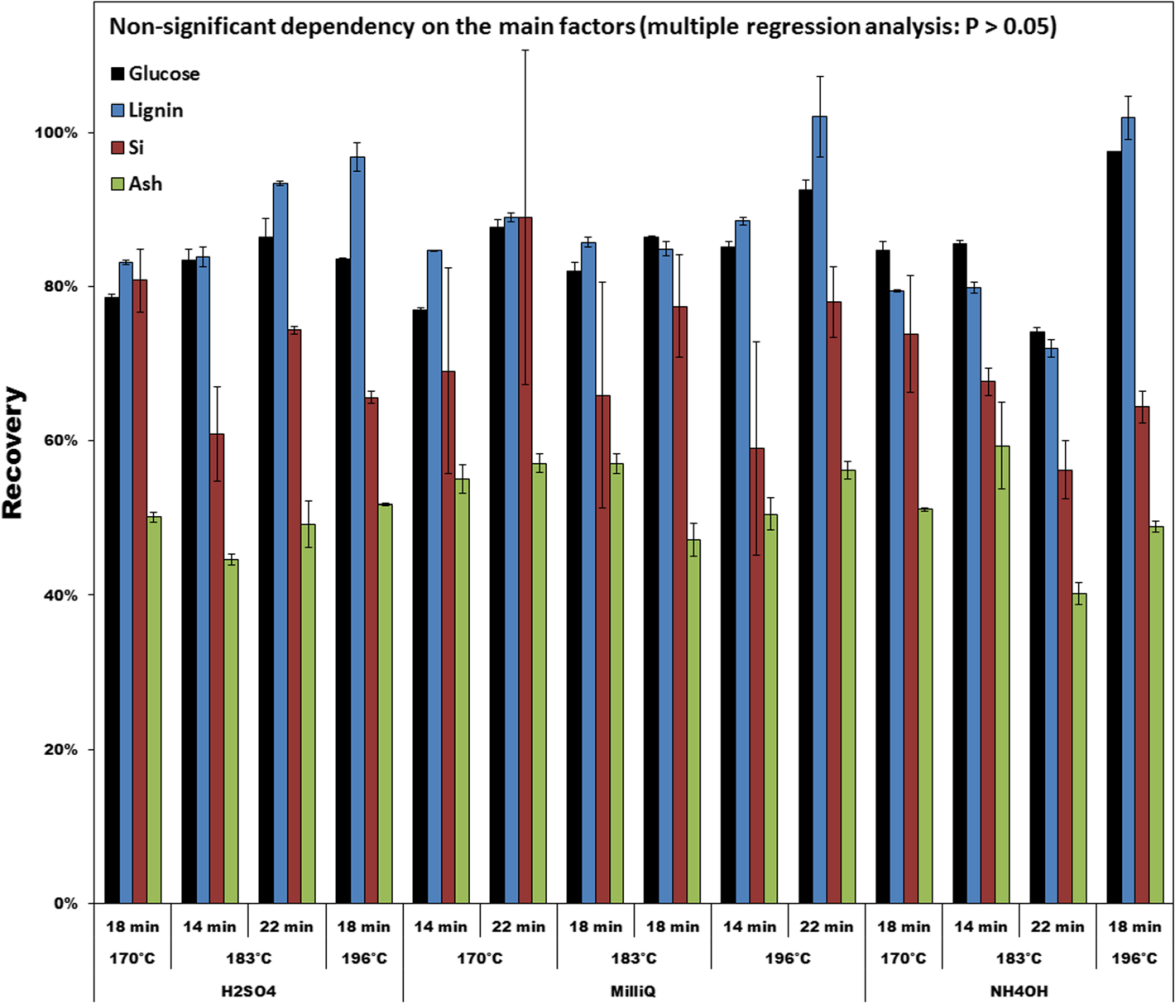
**Recovery of glucose, lignin, silicon (Si), and ash in the washed fiber fraction of hydrothermal pretreated wheat straw (weight base, dry matter).** No dependency of the pretreatment conditions were observed by multiple linear regression (whole model *P* > 0.05).

Recovery of lignin and glucose in the fiber fractions was typically in the range of 80 to 90%. For silicon and ash, the recoveries were in the range of 60 to 80% and 40 to 60%, respectively (Figure 2). For these components, no general trends in response to the pretreatment parameters could be observed, and multiple linear regression revealed no statistically significant dependency on the main factors (*P* > 0.05). Removal of silicon from the fiber fraction explains some of the removal of ash (Figure 2), but removal of potassium is expected to be more important for this, as it was the most abundant mineral element in the untreated wheat straw, and only 4 to 11% was recovered after pretreatment (Table 1).

**Figure 3:**
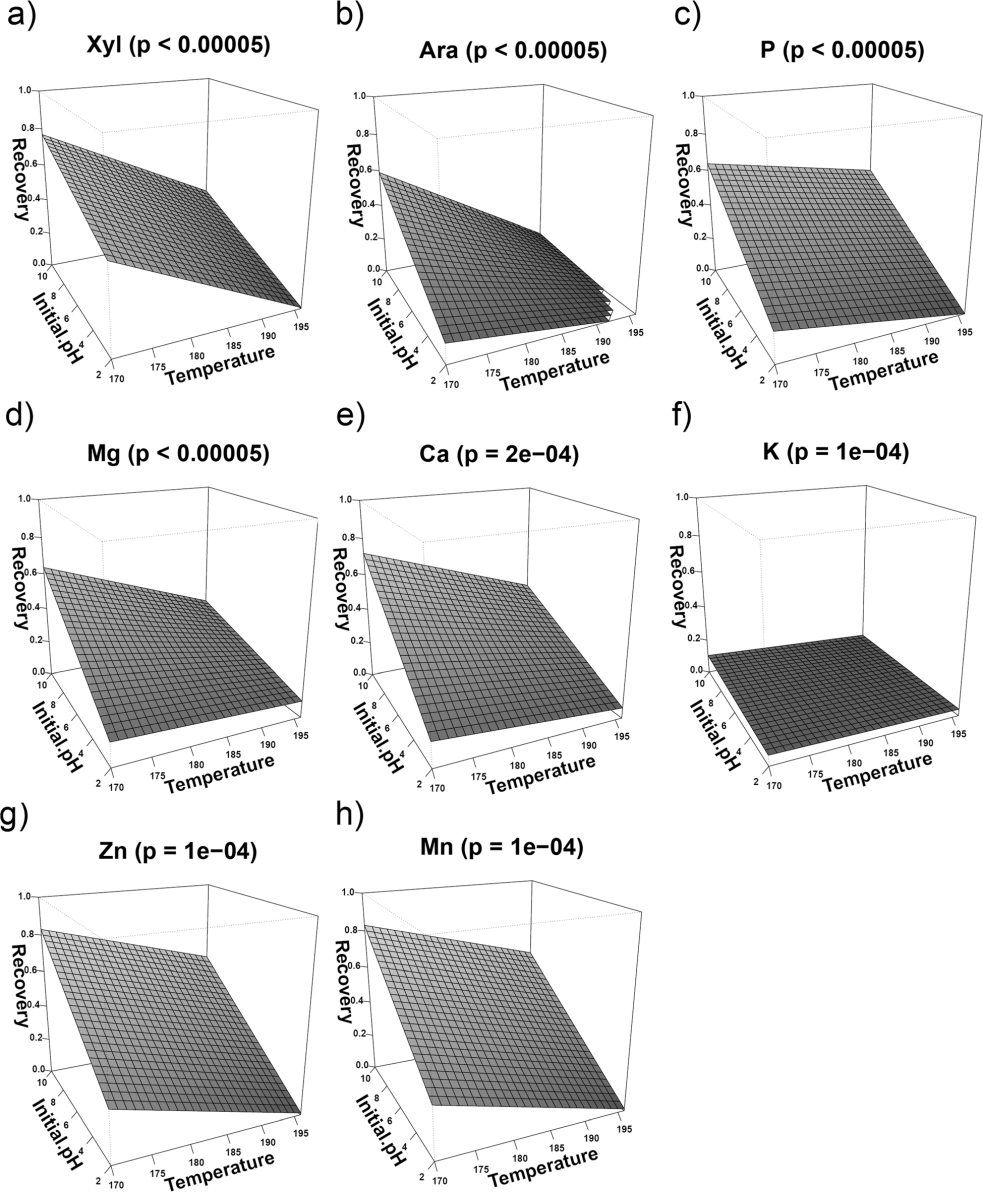
**Response surface modeling of (a) xylose, (b) arabinose, (c) phosphorus, (d) magnesium, (e) calcium, (f) potassium, (g) zinc, and (h) manganese recovered in the washed fiber fraction after hydrothermal pretreatment of wheat straw.** Treatment time was fixed at the center value of 18 minutes, as this factor was non-significant in all models.

By contrast, low pH and high temperatures resulted in reduced amounts of xylose, arabinose, phosphorus, magnesium, and calcium recovered in the washed fiber fraction (Figure 3). The response surfaces of arabinose, calcium, and magnesium were similar, showing high recovery at low temperatures and high pH and low recovery at low pH almost independently of the other main factors. Xylose, phosphorus, zinc, and manganese also showed high recovery at high pH and low temperatures, but also showed decreasing recovery at low pH when the temperature was increased (Figure 3). Potassium in general showed low recovery in the fiber fraction under all pretreatment conditions (Figure 3).

Backwards model reduction resulted in no significant interaction terms (*P* > 0.1) for xylose or phosphorus (Table 2), and treatment time was not a significant factor for these constituents (*P* > 0.2). By contrast, the two other main factors, namely pH and temperature, were statistically significant in the models (*P* < 0.005) (Table 2).

**Table 2:** **Estimated coefficients and** ***P*** **-values for the response surface models**

**Factors**	**Whole model** ***P*** **-value** ^**a**^		**Intercept**	**Temperature**	**pH**	**Time**	**Temperature × pH**	**Temperature × time**	**pH × time**
Xylose	2.876 × 10^−6^	Coef.	3.521	−0.017	0.038	−9.56 × 10^−3^	–	–	–
		*P*-value^b^	1.13 × 10^−6^	1.71 × 10^−6^	5.57 × 10^−5^	0.125	–	–	–
Arabinose	3.589 × 10^−6^	Coef.	0.217	−3.12 × 10^−4^	0.423	9.97 × 10^−3^	−2.13 × 10^−3^	–	–
		*P*-value	0.701	0.919	5.83 × 10^−4^	0.038	1.01 × 10^−3^	–	–
Phosphorus	8.009 × 10^−5^	Coef.	1.331	−6.47 × 10^−3^	0.058	0.010	–	–	–
		*P*-value	0.014	0.019	1.55 × 10^−5^	0.213	–	–	–
Magnesium	2.165 × 10^−5^	Coef.	−0.908	3.47 × 10^−3^	0.467	0.017	−1.92 × 10^−3^	–	4.13 × 10^−3^
		*P*-value	0.130	0.249	4.25 × 10^−4^	0.098	4.22 × 10^−4^	–	0.017
Calcium	3.33 × 10^−4^	Coef.	−0.869	1.25 × 10^−3^	0.488	0.035	−1.66 × 10^−3^	–	−7.21 × 10^−3^
		*P*-value	0.396	0.810	0.010	0.064	0.060	–	0.019
Potassium	1.801 × 10^−4^	Coef.	2.51 × 10^−2^	−2.38 × 10^−4^	5.36 × 10^−2^	3.25 × 10^−3^	−2.15 × 10^−4^	–	−5.99 × 10^−4^
		*P*-value	0.782	0.615	3.65 × 10^−3^	0.059	0.014	–	0.028
Zinc	1.594 × 10^−3^	Coef.	1.683	−7.36 × 10^−3^	0.052	6.67 × 10^−3^	–	–	–
		*P*-value	0.018	0.037	3.97 × 10^−4^	0.519	–	–	–
Manganese	4.552 × 10^−4^	Coef.	1.481	−0.011	0.187	0.029	–	–	6.654 × 10^−3^
		*P*-value	0.065	6.32 × 10^−3^	0.018	0.245	–	–	0.092

Coef., coefficient.

^a^Whole model *P*-values are given in the top row. These were calculated by comparing the models to their respective null models using *F* statistics. High *P*-values (*P* > 0.05) indicate no dependency on the model factors. Non-significant models (glucose, lignin, silicon, iron, aluminum, copper) are not shown.

^b^ *P*-values for each factor indicate the statistical significance of this factor in the model, with low values indicating high significance. Non-significant interactions terms (*P* > 0.1) were removed from the model by single-term backwards model reduction.

Two interaction terms involving pH were significant for calcium, magnesium, and potassium, as pH turned out to be the crucial factor for the recovery of these elements in the fiber fraction (Table 2). Treatment time was not a significant main factor, and temperature was significant in the model only through its interaction with pH. The model for arabinose had one significant interaction term (temperature × pH), while treatment time and pH were the only significant main factors (*P* < 0.1) (Table 2).

### Correlation between biomass constituents

Correlation between the recoveries of each constituent in the fiber fraction after hydrothermal pretreatment was studied by principal component analysis (PCA) and cluster analysis. We found that 81.5% of the variation in the data could be explained by the first two PCs. The PCA scores revealed that the first and most important PC was controlled by pH and temperature, while the second PC seemed to be governed by treatment time (data not shown).

**Figure 4:**
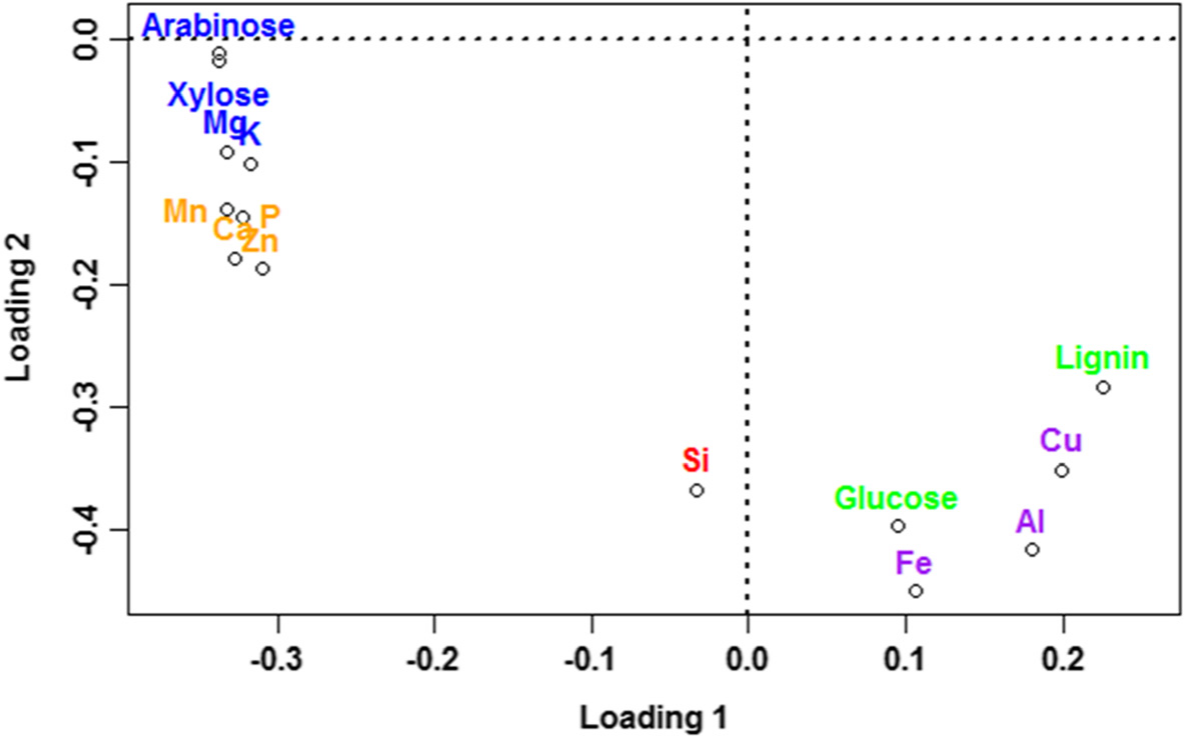
**Loadings plot from principal component analysis (PCA) of the constituents in the washed fiber fraction after hydrothermal pretreatment of wheat straw.** Each constituent is colored according to the cluster to which it belongs, based on the cluster analysis in Figure 5.

**Figure 5:**
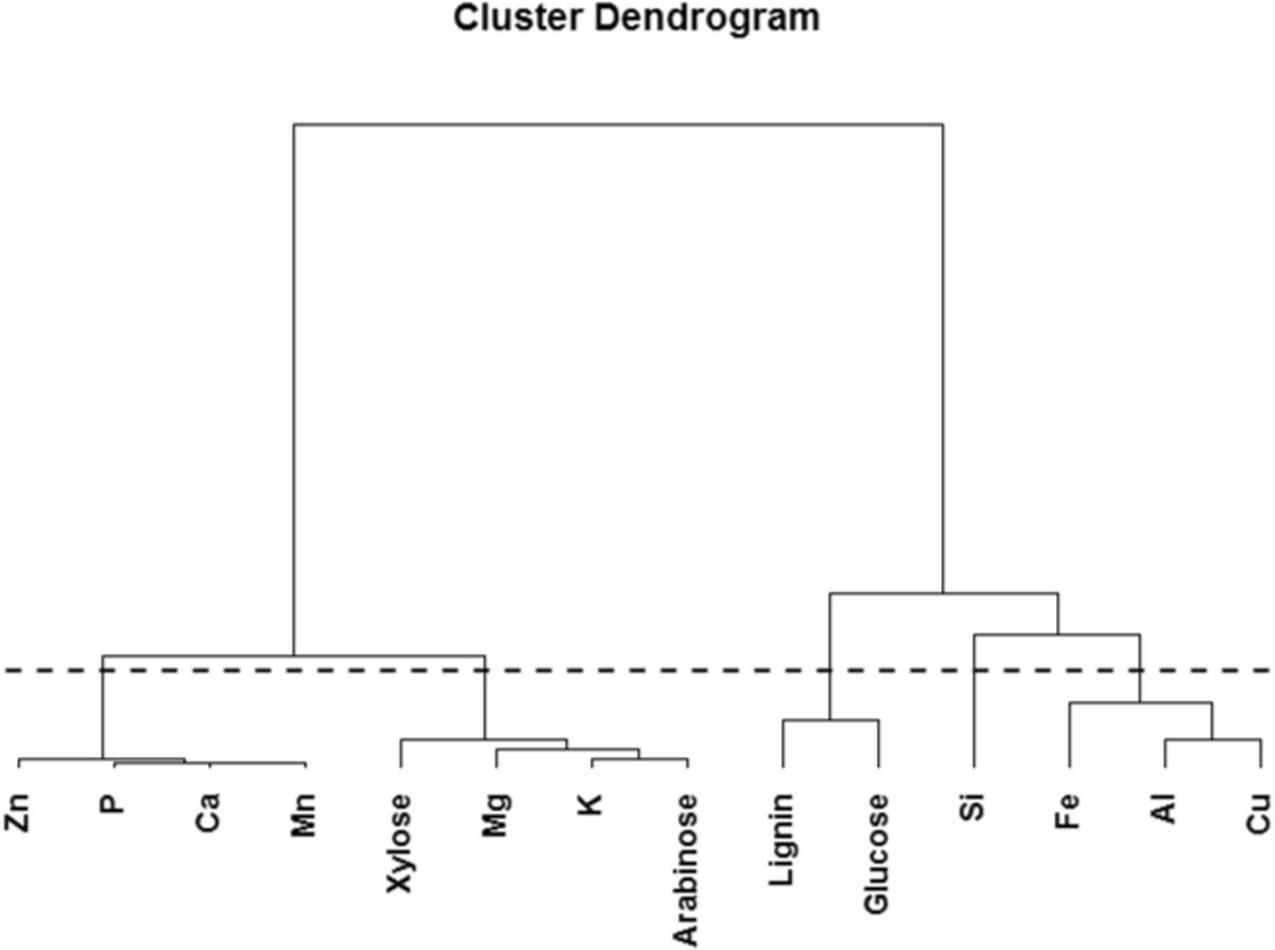
**Cluster analysis of the data used in principal component analysis (PCA), resulting in five clusters.**

The PCA loadings plot with the two first PCs caused separation into two groups: those that were dependent and those that were independent of process conditions during pretreatment. In Figure 4, constituents dependent on process conditions were localized to the top left corner of the plot, and were water-soluble cations and hemicellulosic constituents, such as arabinose and xylose. Constituents independent of process conditions were localized to the bottom right corner, and comprised water-insoluble ions, lignin, and cellulose (Figure 4). Cluster analysis corroborated this separation, but further separated the constituents into a total of five clusters (Figure 5).

The water-soluble components group was divided in two clusters, with little variation along the first PC, which accounted for 55% of the variation in the data. One of the clusters containing the hemicellulosic constituents (arabinose, xylose) and two water-soluble cations (magnesium, potassium) had a lower magnitude for the second loading, meaning that their variation was explained to a greater degree by the first PC compared to the other cluster, which in general had a higher negative value for the second loading (Figure 4). The three clusters in the water-insoluble components group (Lignin, glucose; silicon; iron, aluminum, copper (Figure 5)) were spread in the loadings for both the first and second PC, so the resemblance within clusters could not be described by these two PCs alone.

### Optimization of c_pH_ for prediction of fiber fraction composition

**Figure 6:**
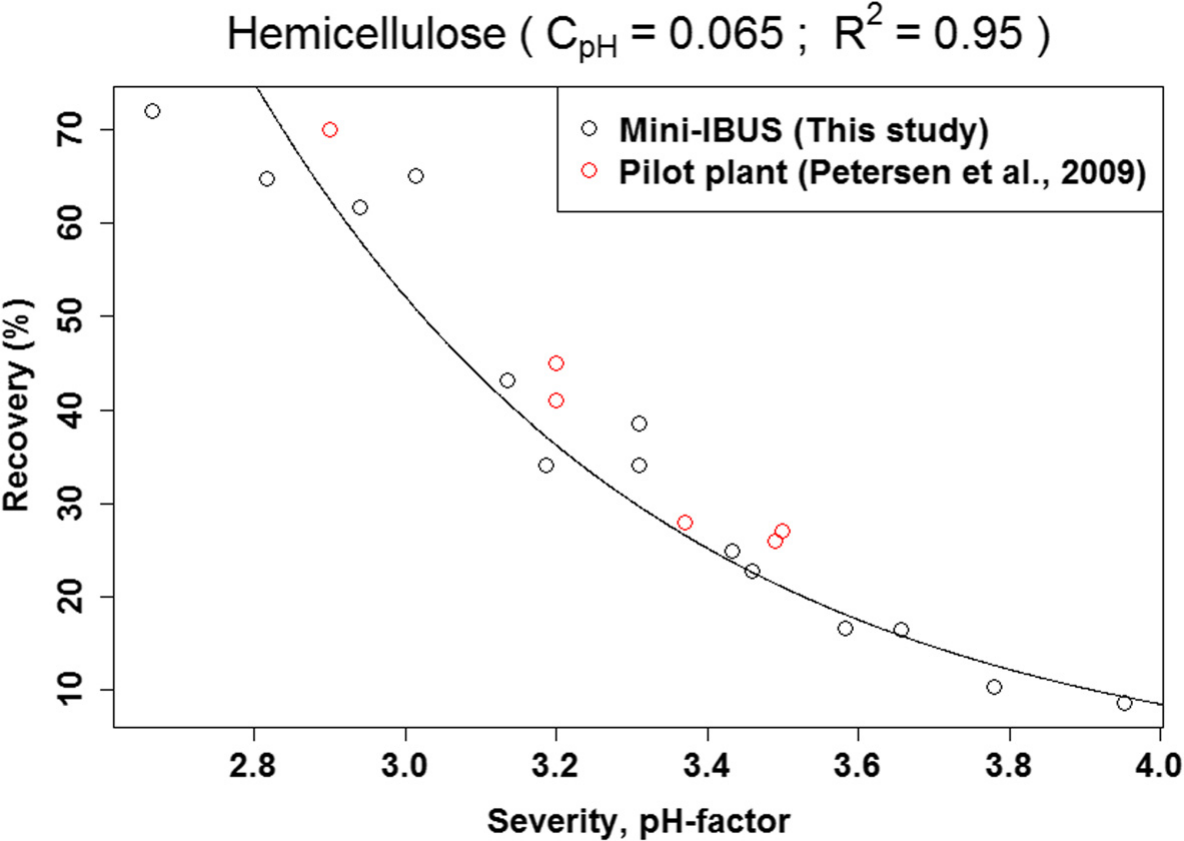
**Recovery of hemicellulose in the fiber fraction in this study compared with the literature [** 13 **], when plotted against the pretreatment-severity with optimized c**_**pH.**_

To predict the composition of the fiber fraction after hydrothermal pretreatment, an extended pretreatment-severity function with an empirical constant, c_pH_, was developed and used. The approach was validated by comparing the model for recovery of hemicellulose in this study with data from similar pretreatments on wheat straw published in the literature (Figure 6).

**Figure 7:**
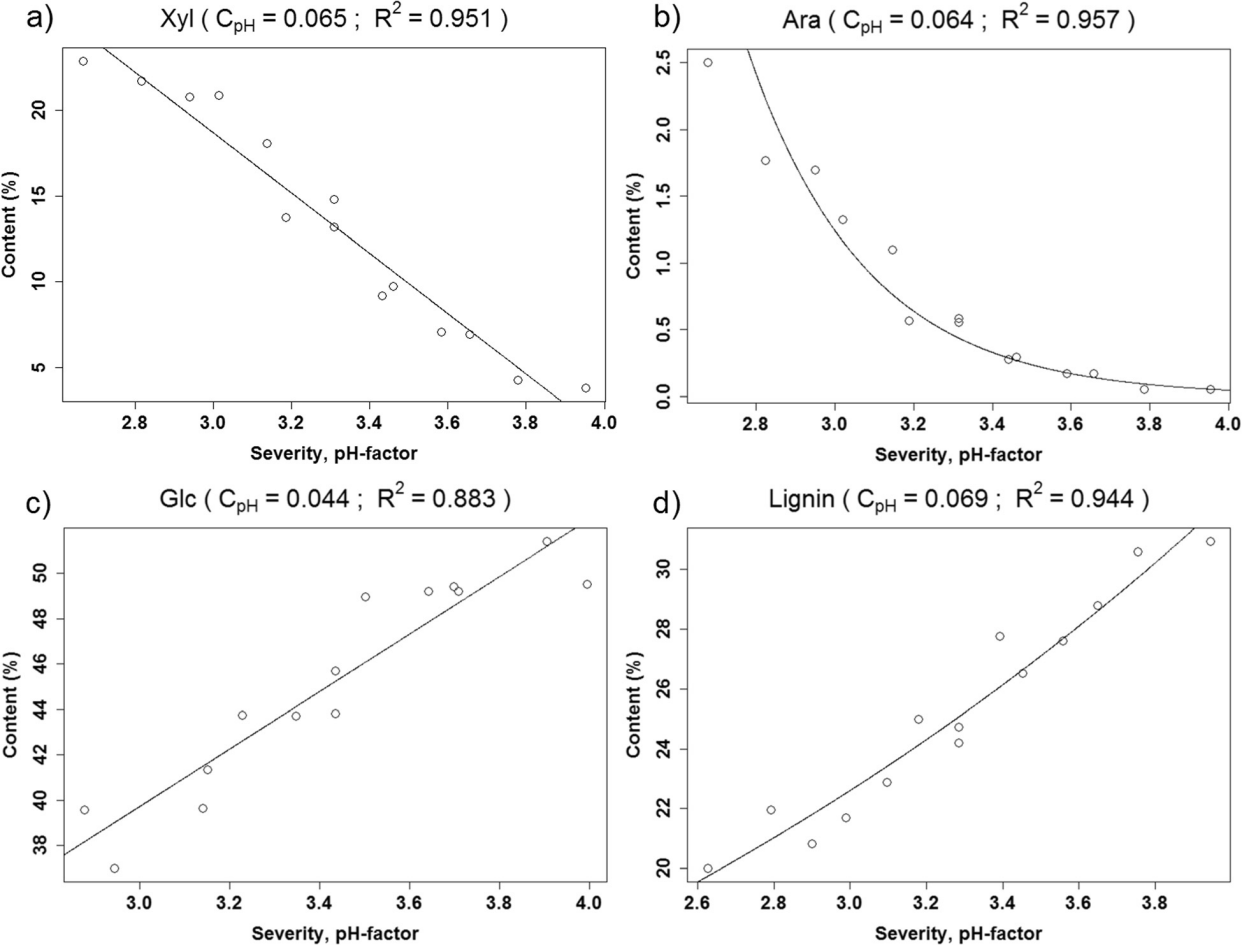
**The experimental data presented as open dots versus the modeled line for the content of (a) xylose, (b) arabinose, (c) glucose, and (d) lignin in the fiber fraction.**

**Figure 8:**
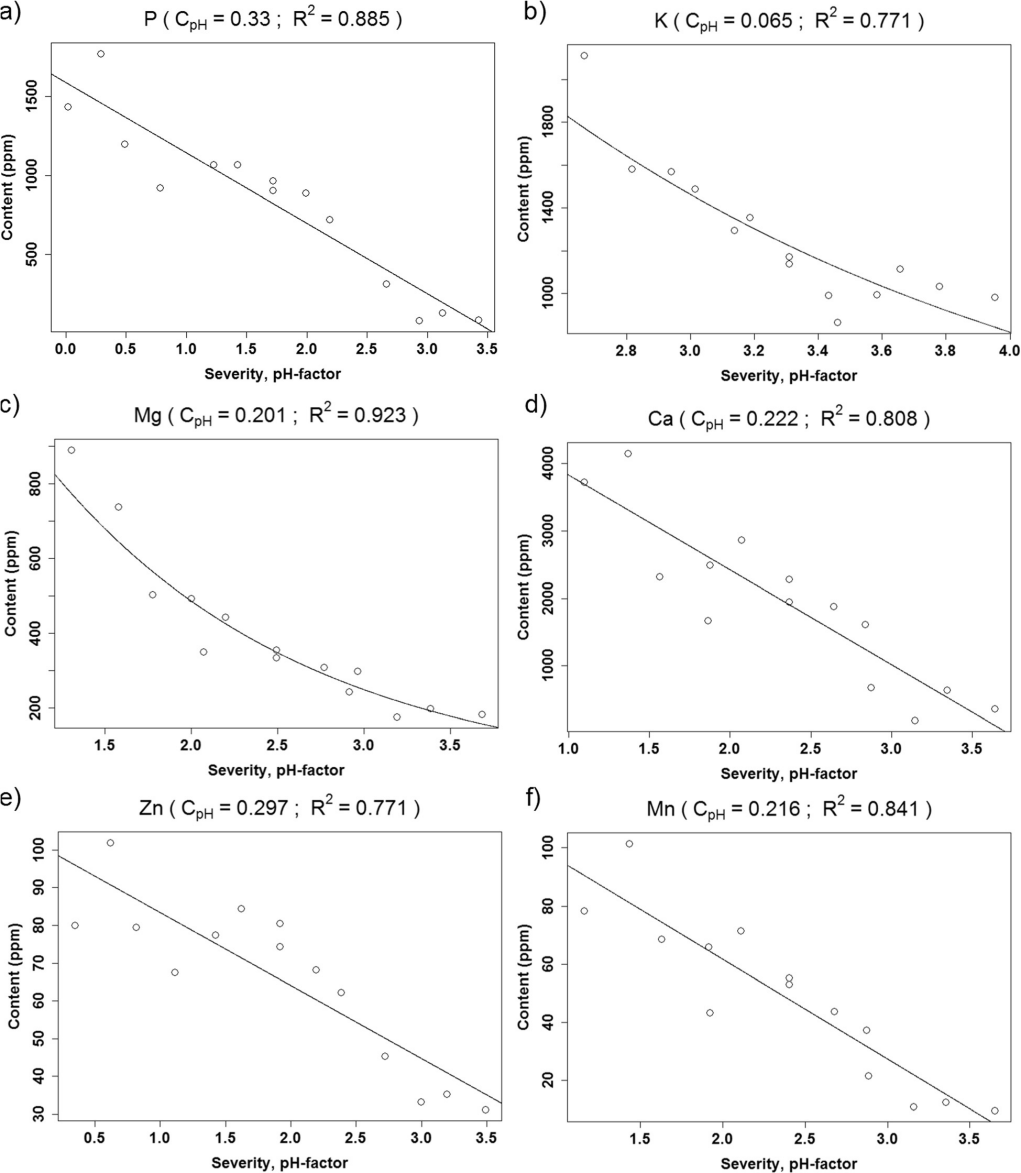
**The experimental data presented as open dots versus the modeled line for the content of (a) phosphorus, (b) potassium, (c) magnesium, (d) calcium, (e) zinc, and (f) manganese in the fiber fraction.**

The optimization of c_pH_ revealed that the pretreatment-severity function could not be generalized for all constituents of the biomass, as some elements were more sensitive to the pretreatment conditions than others. c_pH_ was a direct measure of how sensitive the content of a specific constituent was to pH, as for example, low c_pH_ values resulted in low influence of pH on the pretreatment-severity function (see Eq. (1)). Plotting pretreatment-severity against recovery of each constituent while modifying c_pH_ through an iterative systematic approach, and fitting either a linear or exponential function to each plot, yielded fits of varying R^2^ values. The highest R^2^ value was obtained when the chosen c_pH_ most accurately described the pH sensitivity of the given constituent, so the plot with this c_pH_ value was presented in Figures 7 and 8 for all constituents, where R^2^ > 0.75 could be obtained. Silicon, iron, copper and aluminum had R^2^ values below this threshold. Some data were linear and some were exponential, so it was necessary to fit both types of functions to the data and choose the best fit (highest R^2^).

Lignin and glucose content in the fiber fraction increased with increasing pretreatment-severity, while the xylose, phosphorus, calcium, zinc, and manganese content decreased linearly with pretreatment-severity. The arabinose, potassium, and magnesium content showed an exponential decrease with increasing pretreatment-severity (Figures 7 and 8), whereas silicon, iron, aluminum, and copper levels were insensitive to variation in the pretreatment-severity, as they produced insignificant response models in the design. As observed for the c_pH_ values presented in Figures 7 and 8, the constituents could be divided into two groups: less pH-sensitive constituents (c_pH_ between 0.044 and 0.069) (Figure 7) and more pH-sensitive constituents (c_pH_ between 0.201 and 0.330) (Figure 8). The first group consisted of potassium and the main structural components of wheat straw, that is, glucose, xylose, arabinose, and lignin. The remaining elements (phosphorus, magnesium, calcium, zinc and manganese) constituted the more pH-sensitive group.

## Discussion

### Composition and pretreatment factor analysis

The changes in composition of wheat straw after hydrothermal pretreatment were as expected in terms of xylose, arabinose, and glucose content (Figure 1) [14]. Xylose and arabinose content decreased with high pretreatment-severity, whereas glucose content increased with pretreatment-severity because its recovery was not dependent on pretreatment conditions, and hence, it constituted a larger proportion of the fiber fraction when hemicellulose was solubilized. Ammonium hydroxide has a milder effect than other alkaline solutions (NaOH and KOH) on lignin [15], so recovery of lignin did not increase with increasing pH. As the objective of this study was to investigate the behavior of the mineral components during hydrothermal pretreatment, and to learn about their interactions with the biomass, thus retaining lignin in the fiber fraction was intended. Retention of lignin in the fiber fraction caused minimal variation between samples in terms of the structural components, so that these variations did not overshadow the variations of the less abundant mineral components.

The mineral composition of wheat straw was, in general, in agreement with literature (8850 to 17320 ppm silicon, 50 to 560 ppm aluminum, 70 to 350 ppm iron, 3090 to 4870 ppm calcium, 440 to 660 ppm magnesium, 90 to 190 ppm Na, 4120 to 20720 ppm potassium, 270 to 760 ppm phosphorus) [16]. Some elements were above the stated ranges, but this was expected, because of seasonal and geographical variations. Potassium was the only element solubilized from the fiber fraction under all pretreatment conditions, yielding a low recovery range (Table 1, Figure 3f). This was also as expected, because potassium is exclusively present in the aqueous phase of plant cells, so it is easily leached from the biomass during pre-soaking and pretreatment. Potassium is known to be highly abundant in wheat straw, especially in the cytoplasm and aqueous environments of the vacuole, where it stabilizes the ionic strength of enzymes and osmotic pressure of the cells [17].

Magnesium, in spite of being 70% freely diffusible and present at fairly high concentrations in the cytoplasm, [17] required pre-soaking in acid before it could be solubilized from the fiber fraction (Figure 3d). The same effect of acid pre-soaking was observed for calcium (Figure 3e). Magnesium and calcium are deposited in wheat straw cell walls, where they are associated with carboxyl and phenolic hydroxyl groups of organic components, making them resistant to solubilization [18]. Neither calcium nor magnesium was leached from the relatively intact cell walls (for example, see high recoveries at high pH and low temperatures in Figure 3d). The similarity in results from response surface modeling of calcium and magnesium (Figure 3) to some structural components of the biomass, especially arabinose but also to some degree xylose, indicates that the integrity of the cell wall influences the solubilization of calcium and magnesium.

### Correlation between biomass constituents

Using PCA, it was possible to group the wheat straw constituents into two main groups: water-soluble and water-insoluble constituents (Figure 4).

In the water-insoluble group, lignin and glucose were clustered together. This was not surprising considering that these components interact strongly in lignocellulosic fibers, and are both insoluble across the range of pretreatment conditions tested in this study. Silicon formed a separate cluster, reflecting its unique properties relative to the other elements. Silicon is deposited as SiO_2_ · nH_2_O, either in intimate association with the organic components of plant cell walls or in silica bodies formed within the lumen of specialized cells [19-21]. Owing to the insoluble nature of SiO_2_, even releasing it from the organic material would not remove silicon from the insoluble fraction. Coupled with the high recovery range for silicon in the fiber fraction (Table 1), the implications of these findings are that the vast majority of the silicon is likely to remain associated with lignin and thus accumulate in the lignin residue stream during the further processing of the biomass. Aluminum, iron, and copper were also clustered together. These are all toxic elements for plants if they are accumulated at high concentrations in their free form [18,22,23]. The plants therefore need to control and immobilize these elements to protect themselves from the toxic effects. Aluminum is strongly bound to negatively charged groups in cell walls and is water-insoluble, hence it is not solubilized from the fiber fraction. Iron and copper are also present in an insoluble form in plants [17,18], and are believed to be mainly associated with insoluble cell wall components or phytate [24]. However, some iron in plants is stored in soluble ferritin complexes [25]. As with aluminum and copper, a fraction of the iron was solubilized during hydrothermal pretreatment, but the rest remained in the fiber fraction, regardless of the pretreatment conditions. These findings signify that in relation to biorefining, iron and copper are likely to be distributed between both the aqueous and solid fractions, and to gradually become solubilized during further processing via the enzymatic cellulose and hemicellulose hydrolysis and fermentation steps. Whether such gradual solubilization may function as a nutrient supply during the fermentation, or exert negative effects, warrants further in-depth examination.

In the water-soluble group, arabinose and xylose were located close together in the loadings plot, which was meaningful because in wheat straw they are associated in arabinoxylan. Magnesium and potassium belonged to the same cluster as xylose and arabinose. These two mineral elements are present at relatively high concentrations in the cytoplasm [17], and as the straw matures, they may become loosely bound to negatively charged components within the straw matrix. The present results indicate that magnesium and potassium are unlikely to accumulate in the insoluble fiber streams or in the lignin residue after fermentation in lignocellulose to ethanol processing. The remaining elements (calcium, phosphorus, manganese, zinc) in the water-soluble group belonged to another cluster; their common denominator is that they are all restricted in their movement in plant cells. Calcium and phosphorus interact in calcium–phosphate, calcium–phospholipid, and calcium–phytate complexes [17], and this could explain their similar behavior. Manganese and zinc were found to be present in the fiber fraction at very low concentrations. The low concentrations of manganese and zinc were either due to their low initial abundance in wheat straw, or because they were solubilized during pretreatment, as they exist either as free ions or bound in protein complexes [17]. Manganese and zinc were clustered together with calcium and phosphorus, because the remaining manganese and zinc left in the fiber fraction during hydrothermal pretreatment can interact with the cell wall matrix in a similar fashion to calcium and phosphorus.

### Optimization of c_pH_ for prediction of fiber fraction composition

It is desirable to be able to predict the composition of the fiber fraction based on the severity of hydrothermal pretreatment. The temperature and time dependency of the composition of the fiber fraction was expected to follow the classic pretreatment-severity equation [26], where 14.75 is an arbitrary empirical constant based on the activation energy [27]. The pH dependency varied according to the constituent. Therefore, including pH in pretreatment-severity merely by subtracting pH in the classic method [28] did not result in satisfactory fits; in other words, an additional factor, c_pH_, was needed.

We assumed that there was an underlying dependency of a given constituent on the combined pretreatment-severity, which was linear at low pretreatment-severity, but when pretreatment-severity was increased to a level where most of the constituent was solubilized, leaving no or only strongly restricted residual constituents in the fiber fraction, the dependency was assumed to attain an exponentially decaying nature. Not knowing if the range of pretreatment conditions chosen in this study were in the linear or exponential range for the constituents, we had to fit both a linear and exponential function to the data and choose which of the two gave the best fit (highest R^2^) for each constituent.

As shown in Figure 7b, an exponential function yielded the best fit for arabinose. This was because the pretreatment effectively solubilized arabinose from the fiber fraction, so at high pretreatment-severity the arabinose content approached zero. By contrast, for xylose (Figure 7a), higher pretreatment-severity was needed before an exponential decay could be expected. The magnesium and potassium contents also exhibited an exponential decay, although to a lesser degree than arabinose. Magnesium and potassium were clustered together with arabinose and xylose (Figure 5), and are both elements that occur at high concentrations in the cytoplasm [17]. The remaining magnesium (~10%) and potassium (~4%) recovered in the fiber fraction after hydrothermal pretreatment at high pretreatment-severity might be more recalcitrant to solubilization than the rest, causing exponential decay of their contents at increasing pretreatment-severity. For potassium in Figure 8b, removing the point of low pretreatment-severity, which appeared to be an outlier, still resulted in an exponential decaying function.

The pH dependencies of carbohydrates, lignin, and potassium were lower than those of the other mineral elements, as observed by comparing c_pH_ factors (Figures 7 and 8); c_pH_ values of xylose and arabinose were low. As pH constituted merely a contribution to the severity of pretreatment by opening up the cell wall, it had no direct implications on solubilization of xylose and arabinose. For elements with high c_pH_ (phosphorus, magnesium, calcium, zinc, manganese), low pH could, in addition to opening up the cell wall, increase the solubility of the elements. The solubility of calcium phytate, for example, increases significantly below pH 4 [29].

As seen in Figure 7, contents of glucose and lignin increased with higher pretreatment-severity, because the relative proportion of glucose and lignin increased when xylose and arabinose contents decreased. Variations in glucose and lignin recoveries in the fiber fraction did not depend on the severity of pretreatment, so any change in content must have been governed by removal of other main constituents of the fiber fraction, namely hemicellulose. This explains why the c_pH_ of glucose and lignin were in the range of those of xylose and arabinose.

## Conclusion

By optimizing a factor, c_pH_, indicating pH dependency for each constituent of the biomass, it was possible to model the composition of the wheat straw fiber fraction after hydrothermal pretreatment with respect to xylose, arabinose, glucose, lignin, and mineral elements at varying pretreatment-severities. Solubilization of phosphorus and the mineral elements magnesium, calcium, zinc, and manganese showed high pH dependency. At low pH, these elements were solubilized so that less than 20% by weight compared with the initial amounts present in the untreated wheat straw were recovered in the fiber fraction. At high pH, recovery of these elements was temperature-dependent, presumably due to a combined effect of opening of the cell walls by solubilizing cell wall constituents (mainly hemicellulose) and increased solubility of some elements at acidic pH. The levels of other elements in the fiber fraction, that is, iron, copper, aluminum and silicon, did not depend on pretreatment conditions, and hence could not be modeled.

## Materials and methods

### Wheat straw material

Wheat (*Triticum aestivum L.)* straw was grown and harvested in Denmark in 2012, and cut into pieces approximately 10 cm long prior to hydrothermal pretreatment (see below). The chemical composition of the untreated wheat straw (determined according to National Renewable Energy Laboratory (NREL) procedures [30,31] and multi-elemental analyses respectively (the latter method is described further below) was: 337 g/kg dry matter (DM) glucose, 225 g/kg DM xylose, 30 g/kg DM arabinose, 182 g/kg DM lignin, 57 g/kg DM extractives (fats and proteins), 92 g/kg DM ash, 13.4 g/kg DM potassium, 12.4 g/kg DM silicon, 4.0 g/kg DM calcium, 1.7 g/kg DM phosphorus, 1.1 g/kg DM iron, 1.1 g/kg DM aluminum, 0.9 g/kg DM magnesium, 0.1 g/kg DM sodium, 0.1 g/kg DM manganese,0.1 g/kg DM zinc, and 0.01 g/kg DM copper. Throughout this study, contents of monosaccharides are presented as dehydrated values.

### Hydrothermal pretreatment

Hydrothermal pretreatments were performed in controlled batch runs using Mini-IBUS equipment (Technical University of Denmark, Risø Campus, Roskilde, Denmark). Wheat straw (1 kg DM) was soaked at pH 2, 6 or 10 for 30 minutes, and thereafter treated at 170°C, 183°C or 196°C for 14, 18, or 22 minutes according to a Box-Behnken statistical design with duplicate runs of the center point. pH was adjusted with sulfuric acid and ammonium hydroxide; the concentrations needed to reach the desired pH values of the soaking straw was determined on a small scale prior to the pretreatment campaign.

After hydrothermal pretreatment, the pressure was relieved in the reactor, and the biomass was immediately pressed to 30 ± 4% DM. Afterwards, the fiber fraction was washed in Milli-Q-grade deionized water (1:8 solid:liquid ratio) for 30 minutes at 50°C and 150 rpm, and pressed to 34 ± 5% DM. Liquid fractions were discarded, while all fiber fractions were weighed, frozen ,and then stored at −24°C until further analysis.

### Chemical analysis

Fiber fractions after hydrothermal pretreatment were analyzed for chemical composition by methods based on the standard NREL analytical procedures [30,31]. The analysis of all samples was performed in duplicate with a coefficient of variation (CV) of less 5%, and included DM and ash content determination and strong sulfuric acid hydrolysis for structural carbohydrates and lignin. Untreated wheat straw was subjected to ethanol extraction for 24 hours prior to strong acid hydrolysis because of its high content of extractives (fats and proteins).

### Multi-element analysis

Multi-element analyses of the untreated wheat straw and fiber fractions were conducted by inductively coupled plasma-optical emission spectroscopy (Optima 5300 DV, PerkinElmer, Waltham, MA, USA). To enable silicon analysis with low background values, the sample introduction system was mounted with a hydrogen fluoride (HF)-resistant, silicon-free kit comprising a Dura Mist nebulizer, a Tracey TFE spray chamber, and a Sapphire injector. Prior to analysis, samples (100 mg) were digested at 2,300°C for 25 minutes in a medium consisting of a mixture (v/v) of 47.3% HNO_3_, 4% H_2_O_2_, and 2.65% HF in Teflon tubes in a pressurized microwave oven (UltraWave, Milestone Inc., Sorisole, Italy). The addition of HF ensured that silicon was solubilized and remained in solution during the analysis. Before analysis, samples were diluted to 3.5% HNO_3_ with Milli-Q element water (Merck Millipore). Data quality was evaluated using a certified reference material (spinach; NCS ZC73013, National Analysis Center for Iron and Steel, China), internal standard additions of silicon, and true blanks. Data were processed using WinLab32 software (v3.1.0.0107; PerkinElmer, Waltham, MA, USA). For each element, more than one wavelength was used for analysis to decrease the possibility of matrix interference.

### Statistical analysis

R statistical software (v3.0.2) was used for statistical data analysis [32]. Response surface modeling was performed on the recoveries of constituents in the fiber fraction and presented as perspective plots of the response surfaces. PCA was performed to study and visualize correlations between the constituents of the fiber fractions. Score plots were used to deduce which PCs were governed by which pretreatment factors, while loading plots were used to show the correlation between the different constituents. Cluster analyses were performed by ascendant hierarchical clustering using the ClustOfVar package [33].

To allow prediction of fiber fraction composition after hydrothermal pretreatment, an empirical factor, denoted c_pH_, in an extended pretreatment-severity equation, Eq. (1), was optimized in the interval 0 to 1 to obtain the best linear or exponential fit to the data.

1 $$ \log \left({R}_e\right)= Log\left(t\cdot {e}^{\frac{T-100}{14.75}}\right)-{c}_{pH}\cdot p{H}_{initial} $$

where R_e_ is the extended pretreatment-sevxerity factor, *t* is the treatment time in minutes, *T* is the treatment temperature (°C), and 100 is the reference temperature (°C). 14.75 is a fitted value of an arbitrary activation energy constant (*ω*) when assuming pseudo-first-order kinetics [26,34]. All models were validated by QQ plot of the residuals (data not shown).

## Abbreviations

HF: Hydrofluoric acid
NREL: National Renewable Energy Laboratory
PC: Principal component
PCA: Principal component analysis
